# From Simple to Sinister: Kaposi Sarcoma Masquerading as a Subconjunctival Hemorrhage

**DOI:** 10.7759/cureus.45296

**Published:** 2023-09-15

**Authors:** Nur Syazwani Redzuwan, Nor Azita Ahmad Tarmizi, Safinaz Mohd Khialdin

**Affiliations:** 1 Ophthalmology, Universiti Kebangsaan Malaysia Medical Centre, Kuala Lumpur, MYS; 2 Ophthalmology, Hospital Kuala Lumpur, Kuala Lumpur, MYS

**Keywords:** hiv, aids, kaposi sarcoma, conjunctival kaposi sarcoma, subconjunctival hemorrhage, retrovirus, acquired immunodeficiency syndrome, immunocompromised, kaposi

## Abstract

A young male in his early 30s presented with spontaneous left eye redness for three months associated with blood-stained eye discharge. There was no history of trauma, blood dyscrasias, or anticoagulant intake. On examination, visual acuity was normal in both eyes. An anterior segment examination of the left eye showed subconjunctival hemorrhage with a fleshy bright red conjunctival mass hidden in the inferotemporal fornix. Other parts of the ocular examination including the contralateral eye were unremarkable. Upon further inquiry, the patient revealed a history of a retroviral disease diagnosed eight years ago but had not pursued treatment. Systemic examination revealed a raised non-pigmented lesion of the tongue and a painless purplish plaque at the back. Investigations showed a high viral ribonucleic acid (RNA) load and confirmed the Kaposi sarcoma of the conjunctiva, tongue, and skin; cryptococcal meningitis; smear-negative pulmonary tuberculosis; and late latent syphilis. He was comanaged by multidisciplinary teams. Highly active antiretroviral therapy (HAART) was commenced. Treatment was a challenge considering the simultaneous presence of malignancy and a serious fungal infection of the brain in an immunosuppressed patient. Fortunately, three months post treatment, he showed remarkable improvement as there was almost a complete resolution of conjunctival Kaposi sarcoma. This case revealed an unusual presentation of Kaposi sarcoma affecting the conjunctiva, which could have been mistaken for simple subconjunctival hemorrhage in a young patient without informed comorbidity.

## Introduction

Kaposi sarcoma is a low-grade vascular neoplasm that typically affects individuals with acquired immunodeficiency syndrome (AIDS), immunosuppression, or organ transplantation [1]. There are only a few reported cases of ocular Kaposi sarcoma presenting as an initial manifestation of acquired immunodeficiency syndrome (AIDS) [2]. If Kaposi sarcoma is suspected, prompt investigations should be performed as isolated Kaposi sarcoma may lead to the discovery of AIDS and multifocal Kaposi sarcoma, as occurred in our case.

This article was previously presented as a poster at the 12th Conjoint Ophthalmology Scientific Conference on September 17, 2022.

## Case presentation

A 34-year-old Malay male presented with spontaneous left eye redness that has persisted for three months. Over the past month, it has been progressively worsening and associated with blood-stained discharge. There was no history of blood dyscrasias, trauma, or anticoagulants. The patient also reported experiencing fever, productive cough, loss of appetite, and loss of weight for the past three days. There was no headache, nausea, or vomiting. He strongly denied any prior medical conditions, blood transfusions, tobacco, alcohol, or intravenous use of illegal drugs.

**Figure 1:**
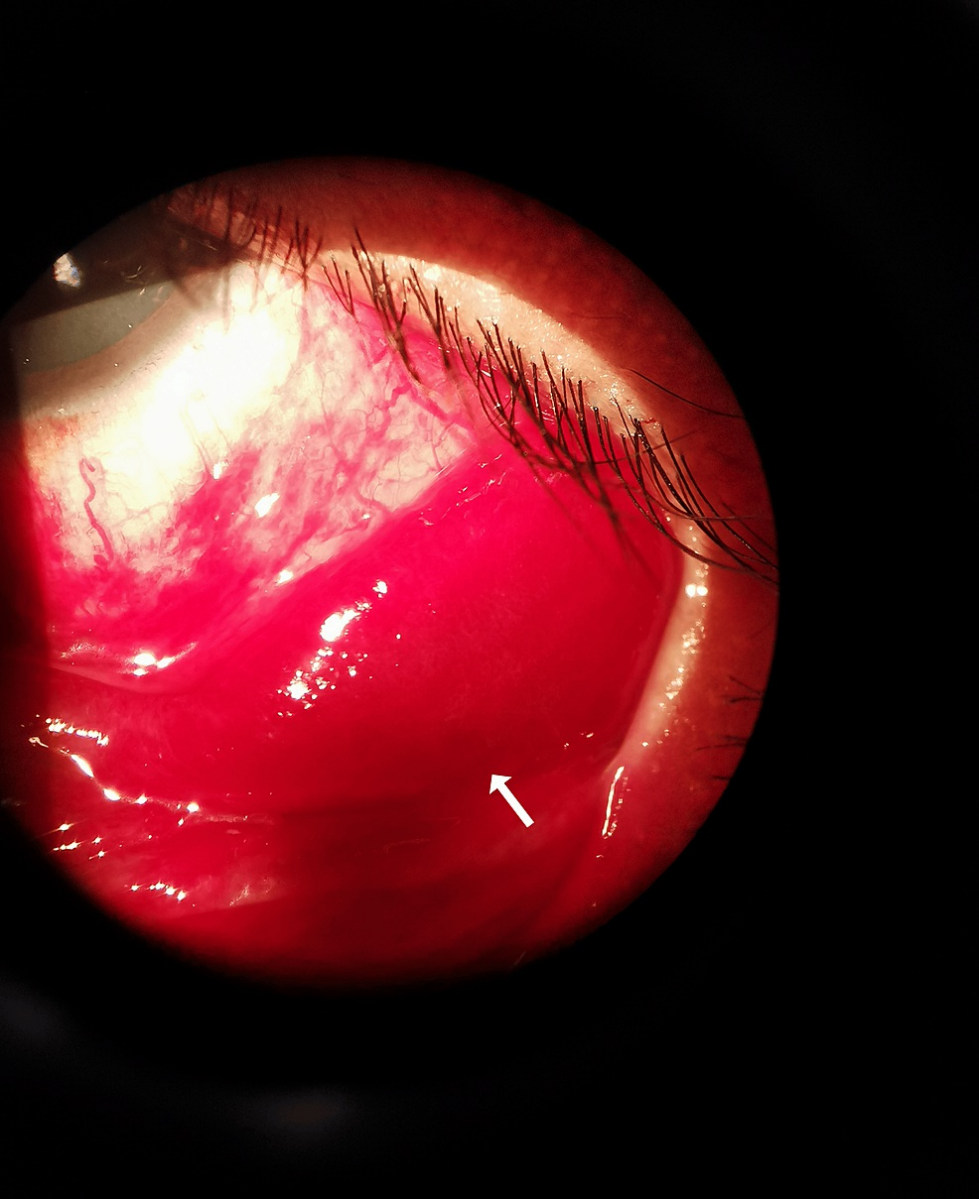
A hidden, vivid red conjunctival mass (arrow) located at the inferotemporal fornix of the left eye

Following an examination, both eyes showed normal visual acuity. However, the left eye exhibited a noticeable subconjunctival hemorrhage inferiorly, accompanied by a concealed conjunctival mass located in the inferotemporal fornix (Figure 1). This conjunctival mass appeared vividly red and fleshy. It was mobile and non-tender on palpation. No feeding vessel, corkscrew vessel, proptosis, or bruit was observed. The patient’s eye movements were unrestricted and painless, and all other aspects of the ocular examination, including the right eye, yielded normal findings.

**Figure 2:**
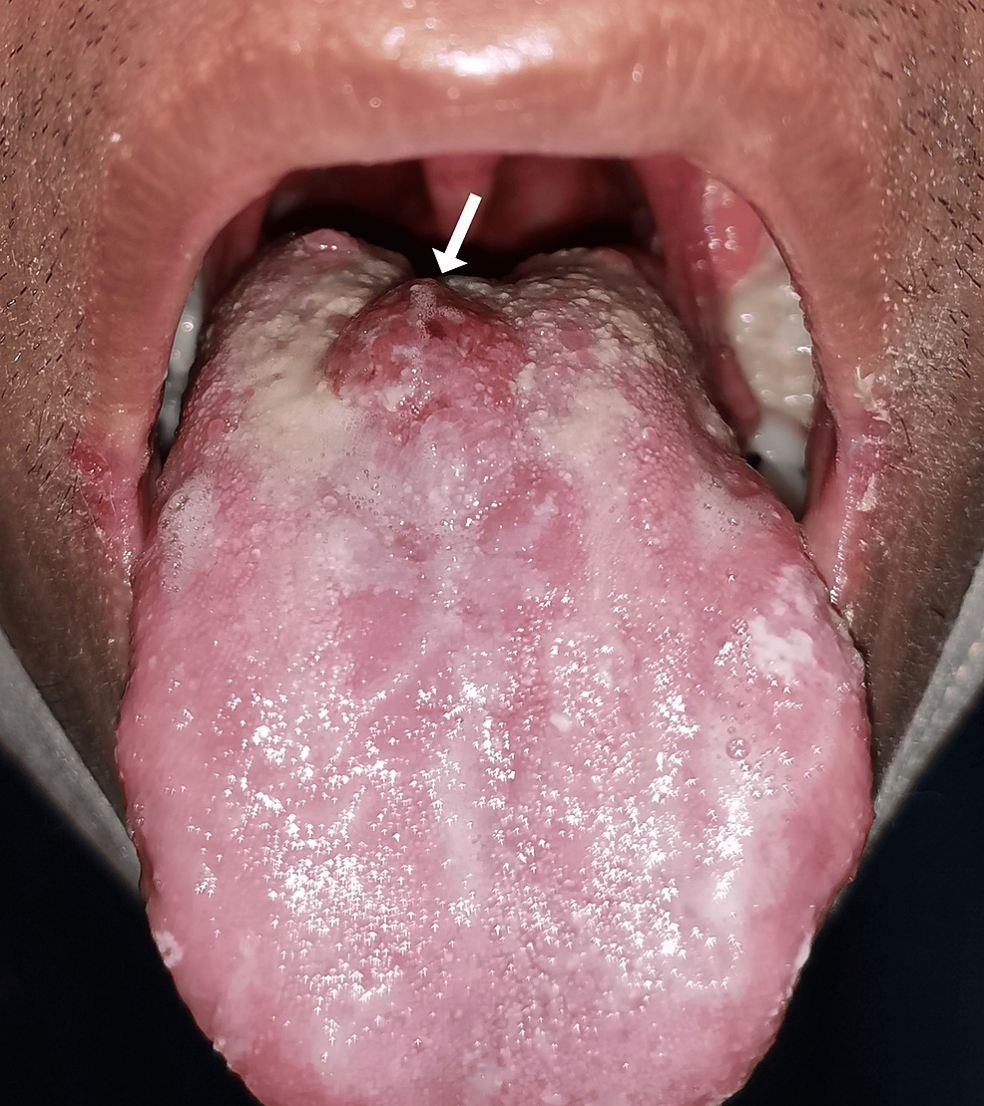
Kaposi sarcoma of the tongue (arrow) with multiple white plaques suggestive of oral thrush

**Figure 3:**
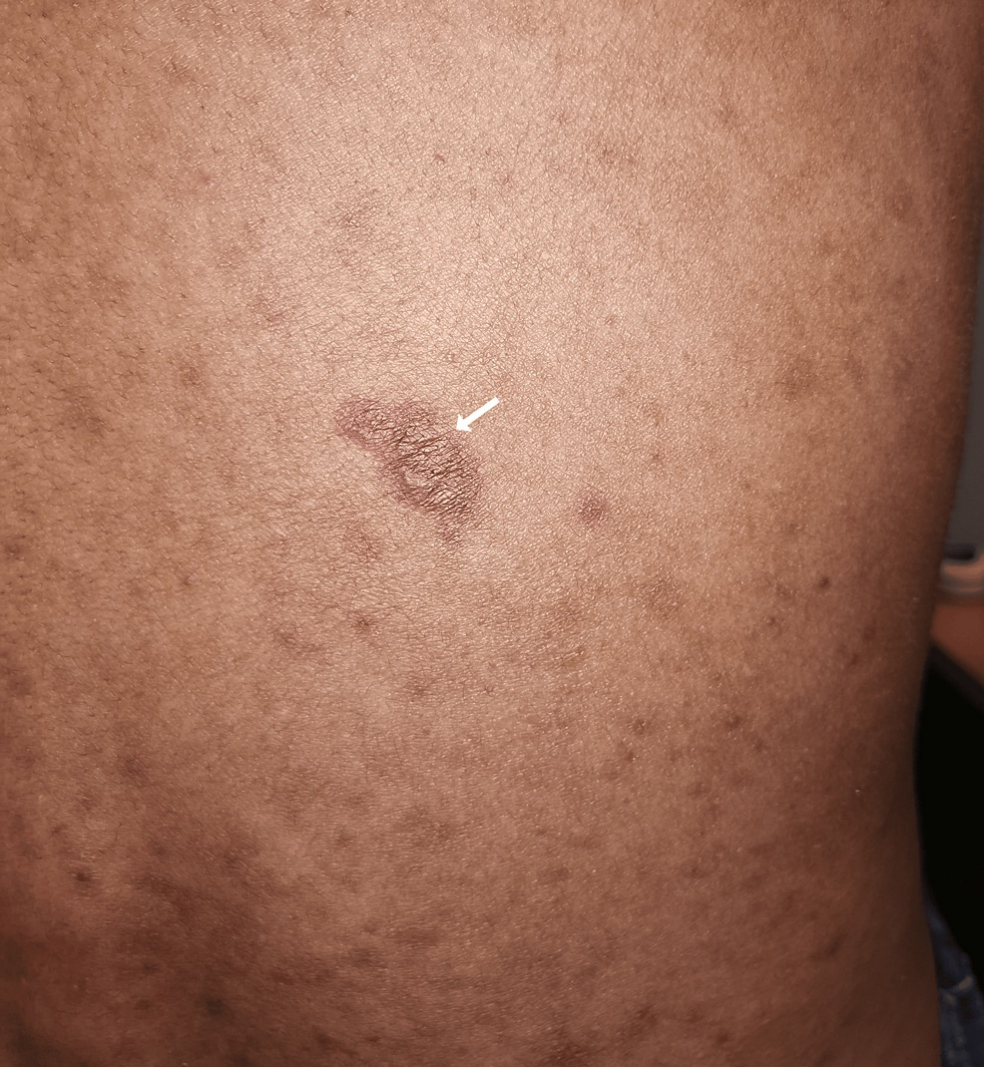
Cutaneous Kaposi sarcoma (arrow) on the posterior trunk

Due to a strong suspicion of conjunctival Kaposi sarcoma, a thorough systemic examination was conducted, which revealed generalized whitish plaque within the oral cavity suggestive of oral thrush with a raised non-pigmented lesion of the tongue (Figure 2), as well as a painless violaceous plaque on his back (Figure 3), further heightening the suspicion.

Consequently, a discussion was initiated with the patient, who eventually admitted to withholding vital medical information. He revealed that he had been diagnosed with acquired immunodeficiency syndrome (AIDS) eight years ago but had not pursued treatment. He admitted that he is currently sexually active with multiple male sexual partners. He disclosed that a few months before seeking medical attention, he was treated for late latent syphilis with three doses of intramuscular benzathine penicillin over three weeks.

He was subsequently admitted to the ward and investigated to confirm his AIDS diagnosis. The infectious disease team was consulted to comanage this patient. His full blood count revealed pancytopenia, with a hemoglobin level of 10 g/dL, a total white cell count of 2.25 × 10^9^/L, and a platelet count of 147 × 10^9^/L. A full blood picture confirmed pancytopenia with no blast cells to suggest acute leukemia. A bone marrow aspiration and trephine biopsy were performed and reported as nonspecific reactive changes associated with human immunodeficiency virus (HIV) infection. His enzyme-linked immunosorbent assay (ELISA) serology for HIV came back positive with an HIV ribonucleic acid (RNA) viral load of 1,540,000 copies/mL. Unfortunately, the cluster of differentiation 4 (CD4) count was unavailable due to unforeseen reasons. There were no concomitant hepatitis B or hepatitis C infections. His rapid plasma reagin (RPR) and *Treponema pallidum* particle agglutination (TPPA) tests were positive, with the RPR showing a titer of 1:64. He was also worked up for tuberculosis. His chest radiography revealed a ground glass appearance in the bilateral lower lung zones, with three negative sputum acid-fast bacilli (AFB) tests. In the absence of exertional dyspnea and negative sputum for AFB, he underwent contrast-enhanced computed tomography (CECT) of the brain, thorax, abdomen, and pelvis that is consistent with pulmonary Kaposi sarcoma, along with metastasis to mediastinal, para-aortic, and inguinal lymph nodes. The cerebrospinal fluid tested positive for *Cryptococcus neoformans,* which was suggestive of cryptococcal meningitis. Other opportunistic infection screening was negative for cytomegalovirus and toxoplasma. A conjunctival biopsy confirmed the diagnosis of Kaposi sarcoma, and subsequent biopsies of both the tongue and skin revealed similar features of the condition.

He was subsequently diagnosed with advanced retroviral disease with cryptococcal meningitis, late latent syphilis, smear-negative tuberculosis, and disseminated Kaposi sarcoma. He was treated with intravenous amphotericin B at a daily dose of 0.7 mg/kg for two weeks followed by oral fluconazole at 400 mg twice daily for eight weeks. Highly active antiretroviral therapy (HAART) was started after four weeks of cryptococcal meningitis treatment. The patient was also empirically treated for smear-negative tuberculosis for a total of six months and received three doses of weekly intramuscular benzathine penicillin at 2.4 million units for his late latent syphilis.

**Figure 4:**
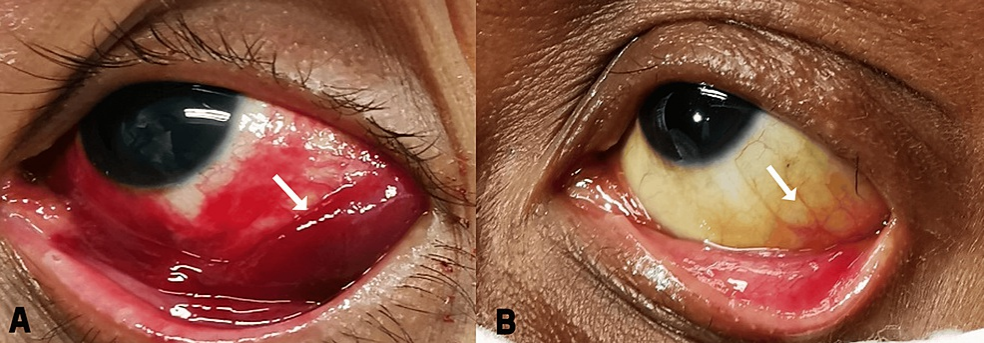
Regression of conjunctival Kaposi sarcoma (arrow) of the left eye (A) Before treatment and (B) after three months of receiving chemotherapy and HAART treatment HAART: highly active antiretroviral therapy

Determining the optimal timing for chemotherapy for Kaposi sarcoma posed a challenge due to the concurrent severe fungal brain opportunistic infection in advanced AIDS. Considering the advanced stage of Kaposi sarcoma, chemotherapy was administered to the patient. This treatment commenced a day after the initiation of HAART and consisted of intravenous paclitaxel and carboplatin, given every three weeks for six cycles. Fortunately, a remarkable improvement of the systemic conditions, including an almost complete regression of conjunctival Kaposi sarcoma, was seen following three months of treatment with HAART and chemotherapy (Figure 4).

## Discussion

Kaposi sarcoma is an indolent angio-proliferative spindle cell tumor associated with human herpesvirus 8 (HHV-8) infection. It usually involves homosexual males with acquired immunodeficiency syndrome (AIDS) [3].

Ophthalmic Kaposi sarcoma may involve the eyelids, conjunctiva, caruncle, and lacrimal sac. The eyelid involvement of Kaposi sarcoma is seen in 6%-16% of ophthalmic Kaposi sarcoma cases, whereas 7%-18% of cases are seen in the conjunctiva, predominantly in the inferior conjunctiva and fornix. Clinically, they present as a painless, red-purple to bright red nodular mass [4] that may masquerade as other conditions such as subconjunctival hemorrhage, conjunctivitis, episcleritis, scleritis, melanosis, malignant melanoma, squamous cell carcinoma, and cavernous hemangioma [5].

If orbital or disseminated Kaposi sarcoma is suspected, imaging studies may be performed. Otherwise, it is usually not required. For a definitive diagnosis of the mass, a biopsy of the lesion may be required. A histopathological examination of the lesion demonstrates vascular channels and proliferating spindle cells surrounded by inflammatory cells [4].

The treatment of acquired immunodeficiency syndrome-related Kaposi sarcoma includes immune reconstitution with highly active antiretroviral therapy (HAART). Kaposi sarcoma tends to respond to chemotherapy [4]. A combination of HAART and chemotherapy has been reported to increase the response rate to 50%-82% in advanced Kaposi sarcoma cases [6]. In cases with progressive or extensive involvement of this disease, systemic chemotherapy is used, whereas in focal ocular lesions, surgical resection, radiation, cryotherapy, or intralesional chemotherapy may be opted for [2]. However, most lesions require observation and regular monitoring only [4].

## Conclusions

This case highlighted a rare manifestation of Kaposi sarcoma affecting the conjunctiva, a condition that might have been incorrectly identified as a common subconjunctival hemorrhage in a young patient without prior knowledge of underlying comorbidities. The accurate identification of conjunctival Kaposi sarcoma played a pivotal role in uncovering an HIV infection, as well as several other opportunistic infections. Without timely intervention, these infections could have led to severe consequences, potentially culminating in a life-threatening event. Therefore, early detection and intervention played a critical role in ensuring the patient’s well-being.
